# Editorial: Women in biofilms vol. II

**DOI:** 10.3389/fcimb.2023.1271026

**Published:** 2023-08-17

**Authors:** Carolina H. Pohl, Angela França

**Affiliations:** ^1^ Department of Microbiology and Biochemistry, University of the Free State, Bloemfontein, South Africa; ^2^ Laboratório de Investigação em Biofilmes Rosário Oliveira, Centre of Biological Engineering, University of Minho, Braga, Portugal

**Keywords:** women in science, biofilms, biofilm control strategies, biofilm formation, clinical biofilms

Following the success of the first *Women in biofilm* Research Topic, it is important to provide an additional opportunity for women involved in various aspects of biofilm research to publish their work. Female authors in this Research Topic contribute to the 33% of female researchers in STEM subjects worldwide (UNESCO, 2021) and have made significant contributions to work on biofilms, ranging from the development of novel methodologies to novel antibiofilm agents.

Biofilms are formed by many microbes including Archae, Bacteria (Penesyan et al., 2021) and microbes belonging to the Eukarya (Brake and Hisiotis, 2010). These multicellular structures play important roles in microbial ecology in hosts as well as the environment (Davey and O’Toole, 2000), with current estimates indicating that 80% of prokaryotes form biofilms (Penesyan et al., 2021). It is also true that biofilms often consist of more than one species, including members of different domains such as yeasts and bacteria, and *Candida albicans* and *Streptococcus mutans* (Pohl, 2022). This preferred mode of growth has many implications for the biology of the microbes, including their interaction with the abiotic environment (Brake and Hisiotis, 2010; Penesyan et al., 2021), the host (in the case of commensal or pathogenic microbes) (Vestby et al., 2020), as well as for antimicrobial resistance (Pierce et al., 2013; Bowler et al., 2020).

Various models have been developed for the high throughput study of the growth, biology and inhibition of biofilms. Although the two most common approaches are the microplate method and the Calgary biofilm device, they do have certain limitations. The paper by Zaborskytė et al. provides a flexible and reusable model for biofilm formation. This 3D-printed FlexiPeg system was validated using *Escherichia coli* and *Klebsiella pneumoniae* biofilms and proved to be a simple, low cost and relevant model for the study of these bacterial biofilms.

The interaction between *C. albicans* and *S. mutans* was studied further in the paper by Wu et al. who expanded on their previous work that showed that extracellular vesicles of *S. mutans* increase the ability of *C. albicans* to form biofilms (Wu et al., 2020). In this new study, they show that the vesicles also stimulate *C. albicans* carbohydrate metabolism and dentin demineralization, which may lead to increased caries formation.

Since biofilms pose an increased risk of infection (due to their inherent antimicrobial resistance and antiseptic resistance), it is important to select appropriate antiseptics, although this remains a clinical challenge. The work by Paleczny et al. investigated commonly used antiseptics containing low concentrations of chlorine-based/releasing agents as antibiofilm treatment options. This was done using a range of biofilm models including several Gram-negative and Gram-positive bacteria, as well as *C. albicans*. They showed that the more complex biofilm models are, the better they reflect real-life scenarios, producing biofilms with greater antiseptic tolerance although they also show greater variance. However, the most important finding relates to the use of antiseptics with low chlorine concentrations. They found that the observed antimicrobial action of these antiseptics is not due to inherent activity against microbes, but rather due to the rinsing effect obtained during application.

One strategy explored during the search for new antibiofilm agents is drug repurposing and modification of existing drugs, for example non-steroidal anti-inflammatory drugs (NSAIDs) (Leão et al., 2020). This approach was adopted by Dumitrascu et al. who synthesized and characterized new carbazole derivatives based on the NSAID carprofen. They found that one of these derivatives could inhibit Gram-positive planktonic and biofilm growth and another was active against the Gram-negative *Pseudomonas aeruginosa*.

This Research Topic echoes the sentiment expressed by Almeida and Bakaletz (2022) and presents additional examples of the excellent work performed by women in the study of biofilms of bacteria and yeasts.

## Author contributions

CP: Writing – original draft. AF: Writing – review & editing.
